# Effects of Intravitreal Aflibercept on the Systemic Insulin-like Growth Factor-I and Vascular Endothelial Growth Factor-A in Patients with Diabetic Retinopathy and Age-Related Macular Degeneration

**DOI:** 10.1155/2021/7058505

**Published:** 2021-12-15

**Authors:** Anna Lena Huber, Reinhard Angermann, Yvonne Nowosielski, Christof Seifarth, Martina T. Kralinger, Claus Zehetner

**Affiliations:** ^1^Department of Ophthalmology, Medical University Innsbruck, Innsbruck, Austria; ^2^Department of Ophthalmology, Paracelsus Medical University Salzburg, Salzburg, Austria

## Abstract

**Purpose:**

To analyze the effect of intravitreal aflibercept injections on systemic levels of insulin-like growth factor-1 and vascular endothelial growth factor-A in patients with diabetic retinopathy and age-related macular degeneration.

**Methods:**

Vascular endothelial growth factor-A and insulin-like growth factor-1 levels were determined before and one week and four weeks after intravitreal injection of aflibercept (2.0 mg/50 *μ*l) for 19 patients with age-related macular degeneration (mean age, 76 ± 11 years) and 18 patients with diabetic retinopathy (mean age, 64 ± 14 years). Twenty-two healthy individuals were enrolled as controls.

**Results:**

A significant decline in systemic vascular endothelial growth factor-A level, from 43 (30–57) pg/ml at baseline to 8 (8–8) pg/ml (*p* < 0.001) at week one and 17 (8–25) pg/ml (*p*=0.0054) at week four, was observed in the age-related macular degeneration group. In the diabetic retinopathy group, vascular endothelial growth factor-A levels declined from 53 (35–117) pg/ml to 2 (1–5) pg/ml (*p* < 0.0001) one week after injection and 16 (13–22) pg/ml four weeks after injection (*p*=0.0327). At baseline, systemic insulin-like growth factor-1 concentration was higher in the diabetic retinopathy group (57 [37–99] pg/ml) than in the age-related macular degeneration group (35 [24–51] pg/ml) (*p*=0.0056). A subgroup analysis showed that patients in the proliferative diabetic retinopathy subgroup had significantly higher systemic insulin-like growth factor-1 concentrations (71 [44.7–243] pg/ml) than those in the nonproliferative diabetic retinopathy subgroup (43 [29–66] pg/ml) (*p*=0.0048).

**Conclusions:**

The difference between the baseline systemic insulin-like growth factor-1 levels of the age-related macular degeneration and diabetic retinopathy groups and the higher insulin-like growth factor-1 levels in the proliferative diabetic retinopathy subgroup one week after aflibercept therapy suggest that insulin-like growth factor-1 may play a role in the pathomechanism of diabetic retinopathy.

## 1. Introduction

Diabetic retinopathy (DR) and age-related macular degeneration (AMD) are the most common causes of vision impairment among people in industrialized countries [1–3]. Although numerous risk factors leading to the development of DR [4, 5] or neovascular AMD (nAMD) [6] have been proposed, the etiologies of both diseases are still not fully elucidated. Both diseases are related to the presence of a complex inflammatory process that involves the overexpression of proangiogenic factors, which ultimately leads to choroidal neovascularization and consecutive loss of vision [7, 8]. Among the various proangiogenic factors, vascular endothelial growth factor (VEGF) plays a major role in the development of neovascularization, and until now, anti-VEGF drugs are the only approved therapy for the treatment of AMD [9] and the most efficient therapeutic approach for DR. It is also known that insulin-like growth factor (IGF) is involved in the regulation of neovascularization. Moreover, it plays a role in the expression of VEGF. Therefore, IGF potentially participates in the pathogenesis of diseases associated with pathological angiogenesis [10, 11].

The results of previous studies demonstrate that patients with intermediate AMD and nAMD have increased systemic IGF-1 levels, indicating that IGF-1 is involved in the development of pathological neovascularization [12, 13]. However, evidence regarding the involvement of IGF in the pathogenesis of DR remains inconclusive. Although a cross-sectional study by Payne et al. [14] did not show a difference between the serum IGF-1 levels of participants in a control group and those of patients with DR of various stages, a more recent study by Zhang et al. [15] demonstrated that patients with DR have higher levels of serum IGF-1, thus emphasizing that IGF is a potential contributor to the pathogenesis of DR.

We conducted this study to further elucidate the potential role of IGF-1 and VEGF in the development of pathological neovascularization and vascular leakage of DR and nAMD. Thus, we analyzed and compared the effect of intravitreal injections of aflibercept on systemic levels of IGF-1 and VEGF-A in patients with DR and nAMD. To the best of our knowledge, this is the first prospective study of the systemic response of IGF-1 levels after treatment with intravitreal anti-VEGF therapy for nAMD and DR.

## 2. Materials and Methods

### 2.1. Subjects

This prospective study was performed in accordance with the tenets of the Declaration of Helsinki. Written informed consent for participation was obtained from all patients. Approval and informed consent procedures were approved by the institutional review board of the Medical University Innsbruck (Innsbruck, Austria, Nos. 1261/2020 and 1049/2021).

Seventeen patients with type 2 diabetes mellitus (DM) and diabetic macular edema (DME) and 19 patients with nAMD were included in this study. Each participant received intravitreal injections (IVI) of aflibercept (2.0 mg, 50 *μ*L) for three consecutive months as part of their treatment. None of the patients had received any intravitreal anti-VEGF or corticosteroid therapy within the previous 6 months. All patients with nAMD and DR were diagnosed by a retina specialist after fundus examination and optical coherence tomography (OCT) and either OCT- angiography (Heidelberg Spectralis® OCT-A, Heidelberg Engineering, Heidelberg, Germany) or fluorescein angiography. Patients with DR were classified according to the International Clinical Disease Severity Scales for DR and DME [10].

The exclusion criteria were bilateral disease in need of concurrent therapy, a history of vitrectomy or uveitis, inflammatory comorbidities, systemic vasoproliferative disorders, treatment with anti-inflammatory medications, and previous systemic anti-VEGF therapy. Patients with signs of both AMD and DR were excluded. The control group comprised 22 healthy individuals.

### 2.2. Blood Sample Collection

Blood samples were drawn within 1–3 h before the first IVI, as well as one week and 4 weeks after the first IVI. Blood samples were collected into dipotassium-ethylenediaminetetraacetic acid tubes. Centrifugation was performed at 3000 rpm for 20 min, and plasma was stored within 2 h of collection at −80°C until it was subjected to enzyme-linked immunosorbent assay (ELISA).

### 2.3. Analysis of Systemic VEGF and IGF-1 Levels Using ELISA

Systemic VEGF and IGF-1 levels were determined using ELISA (Quantikine VEGF ELISA Kit; R&D Systems Europe, Abingdon, OX14 3NB, UK; #DVE00 and DG100, respectively) as described by the manufacturer.

### 2.4. Statistical Analyses

All statistical analyses were performed using SPSS Statistics 24® (IBM, Armonk, NY, USA) and GraphPad Prism 8.0.0, for Windows (GraphPad Software, San Diego, CA, USA). The Kolmogorov–Smirnov test was used to test for normal distributions. Continuous data that were not normally distributed were reported as medians with interquartile ranges (IQR). Pearson's or Spearman's rank correlation coefficients were calculated to analyze the correlations between parameters. Categorical data were compared between the treatment groups using the chi-square and Fisher's exact tests. The Friedman test was used to compare the baseline values with the values recorded at one and four weeks after aflibercept injection. Comparison of the baseline values of the control, AMD, and DR groups was performed using the Kruskal–Wallis test, followed by Dunn's post hoc test. A two-way analysis of variance was applied to assess the effects of proliferative diabetic retinopathy (PDR) versus nonproliferative diabetic retinopathy (NPDR) in patients with diabetes. Comparisons of groups were corrected for multiple testing using the Holm–Sidak method. Linear regression models were fitted using standard parameters to identify the factors that influence baseline IGF-1 and VEGF values. Statistical significance was set at a two-sided *p* value <0.05.

## 3. Results

### 3.1. Demographic Parameters

The demographic parameters of the patients in the three groups (control, DR, and AMD groups) are presented in Table 1. The patients with DR were younger than patients in the other groups (*p*=0.0043).

### 3.2. Systemic VEGF-A Concentrations

**Figure 1:**
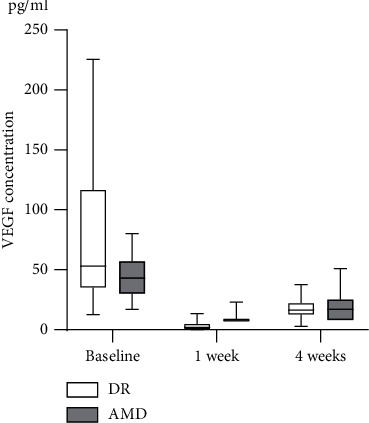
Systemic VEGF-A levels of patients with AMD or DR at baseline and at one week and four weeks after aflibercept administration.

The median (IQR) systemic concentration of VEGF-A in the healthy control group was 61 (38–112) pg/ml compared to 53 (35–117) pg/ml in the DR group and 43 (30–57) pg/ml in the AMD group (Figure 1).

A linear regression model adjusted for age and sex showed that the patients' diseases were the only factors that influenced baseline systemic VEGF-A levels. In the AMD group, the systemic VEGF-A level decreased from a median (IQR) of 43 (30–57) pg/ml at baseline to 8 (8–8) pg/ml (*p* < 0.0001) one week after IVI of aflibercept and remained significantly decreased at 17 (8–25) pg/ml (*p*=0.0054) four weeks after injection. In the DR group, the VEGF-A levels declined from 53 (35–117) pg/ml to 2 (0–5) pg/ml (*p* < 0.0001) one week after injection and remained significantly decreased at 16 (13–22) pg/ml four weeks after injection (*p*=0.0327) (Table 2).

### 3.3. Systemic IGF-1 Concentrations

The median (IQR) systemic concentration of IGF-1 in the healthy control group was 49 (43–61) pg/ml, 57 (37–99) pg/ml in the DR group, and 35 (24–51) pg/ml in the AMD group. A linear regression model adjusted for age and sex showed that the patients' diseases were the only factors that influenced baseline systemic IGF-1 level. There was no significant change in systemic IGF-1 levels within and across the groups after intravitreal aflibercept injection.

**Figure 2:**
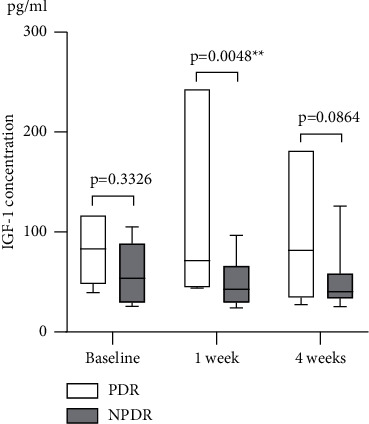
Systemic IGF-1 levels of patients with PDR or NPDR at baseline and at one week and four weeks after aflibercept administration.

A subgroup analysis of the DR group revealed that the IGF-1 level of the PDR subgroup (*n* = 7) was 83 (48–117) pg/ml at baseline and 71 (45–243) pg/ml at one week and 82 (34–181) pg/ml at four weeks after aflibercept administration (*p* > 0.05). IGF-1 levels in the NPDR subgroup (*n* = 11) did not change significantly at one week and four weeks after intravitreal aflibercept therapy (*p* > 0.05). However, the systemic IGF-1 levels in the PDR subgroup were significantly higher than those in the NPDR subgroup one week after aflibercept administration (*p*=0.0048) (Figure 2).

There was no significant difference between the systemic VEGF-A levels of the PDR and NPDR subgroups at baseline and at one week and four weeks (*p* > 0.05, Holm–Sidak test) after intravitreal aflibercept therapy.

## 4. Discussion

In the present study, we observed a significant decrease in systemic VEGF-A levels in the AMD and DR groups one week and four weeks after intravitreal aflibercept therapy. The baseline IGF-1 level was significantly higher in the DR group than in the AMD group. In a regression model adjusted for age and sex, the disease was found to be the main factor that influenced the differences in systemic IGF-1 levels at baseline. After further subgroup analyses of the DR group, we found that patients with PDR had significantly higher systemic IGF-1 levels than patients with NPDR one week after intravitreal aflibercept administration.

The significant decrease in the systemic VEGF-A of patients with AMD and DR one week and four weeks after aflibercept administration reflects a well-known effect of aflibercept leakage into the bloodstream after IVI [16–18]. The unique pharmacokinetic characteristics of aflibercept might explain its possible off-target effects in the systemic circulation after intravitreal administration. Aflibercept has a high affinity for neonatal Fc (FcRn) [19], which has the ability to actively transport the drug molecule across the blood-retina barrier, leading to high systemic bioavailability. Upon reaching the blood stream, FcRn promotes recycling and prevents the antibody from catabolism, thereby extending systemic exposure of aflibercept. A peak of total systemic level of aflibercept is reached seven days after intravitreal injection and declines steadily afterwards [20]. These specific pharmacodynamic properties underline the possibility of estimating changes in the intraocular angiogenic milieu by investigating off-target effects of the drug in the systemic circulation.

In the present study, the DR group had significantly higher baseline systemic IGF-1 levels than the AMD group. Additionally, we found that the PDR subgroup had higher systemic IGF-1 levels than the NPDR subgroup one week after intravitreal aflibercept therapy. This finding is in accordance with the results of a recent report that demonstrated a correlation between systemic IGF-1 levels and the severity of DR, which led the authors to propose that IGF-1 might play a contributing role in the development of DR [15]. In fact, IGF-1 signaling is directly involved in vascularization through endothelial cell migration and tube formation, as well as inflammatory responses and retinal neovascularization [11]. The effectiveness of IGF-1 in transporting glucose into the cell is enhanced when blood glucose level is elevated [21]. Elevated intracellular glucose promotes neovascularization via the activation of protein kinase C (PKC) [22]. PKC increases the production of extracellular matrix and cytokines and enhances vascular cell proliferation [23]. Furthermore, IGF-1 promotes chemotaxis of retinal endothelial cells, a process in which the endothelial cells migrate from deep layers into the vitreous cavity, facilitated by collagenase formation, which is in turn increased by the presence of IGF-1 [21]. This process ultimately leads to the dissolution of the basement membrane and retinal collagen matrix as a prerequisite for neovascularization [21].

IGF-1 has been found to be upregulated in tumor neoangiogenesis models after inhibition of VEGF receptor antibodies [24]. This counterregulatory upregulation could be interpreted as an adaptive escape mechanism from anti-VEGF therapy.

In a study of preclinical models, IVI of IGF-1 induced arteriolar tortuosity, as well as leakage from retinal vessels, followed by the appearance of microaneurysms [25]. IGF-1 and VEGF-A levels have been shown to be associated with severe complications, such as vitreous hemorrhage and retinal detachment, in patients with PDR [1].

In the present study, the systemic IGF-1 levels of patients in the PDR subgroup were significantly higher than those of patients in the NPDR group one week after aflibercept administration. This could be interpreted as an epiphenomenon of the intraocular proliferative disease activity in PDR. It is also in line with previous clinical findings that demonstrated that high systemic IGF-1 levels are correlated with higher prevalence and severity of DR [13, 14]. The findings of the present study are further supported by the missing process of pathological neovascularization observed in knockout models using IGF-1 receptor suppression [26, 27]. Similarly, patients with genetically low IGF-1 levels show fewer vascular branching points in the retinal vasculature, underlining the critical role of IGF-1 in the development of vascularization [28]. In patients with diabetes, IGF-1 bound to the IGF binding proteins 1 and 2 correlates with the development of proliferative DR. Additionally, worsening of DR has been reported after the use of recombinant human IGF-1 to improve glycemic control in patients with diabetes [29].

The present study has some limitations. First, the patients in the DR group had a lower mean age than those in the control and AMD groups. However, the results of a linear regression analysis adjusted for age and sex proved that the disease entity was the only independent influence on baseline systemic IGF-1 level. Second, the DR subgroup comprised a small number of patients. Future studies with greater focus on a more profound analysis of IGF-1 levels in various stages of DR are needed. Additionally, further studies could evaluate IGF-1 levels during and after completion of the loading dose to evaluate the effect of repeated intravitreal aflibercept injections on this proangiogenic cytokine.

In summary, the present study demonstrates that aflibercept significantly decreases systemic VEGF-A in patients with AMD and DR. The difference between the baseline systemic IGF-1 levels of patients in these groups and higher IGF-1 levels in the PDR subgroup one week after aflibercept therapy suggests that IGF-1 plays a role in the pathomechanism of DR.

## Figures and Tables

**Table 1:** Demographic parameters.

	AMD	DR	*p*	Control	*p*
*N*	19	17	ns (FT)	22	
Sex (f/m)	11/8	6/11	ns (FT)	10/12	>0.05 (CS)
Age (median/mean ± SD)	78/75 ± 10.6	63/63 ± 14.3	0.0072^*∗*^	77/75 ± 7.6	*p*=0.0043^*∗∗*^ (KW) *p*=0.0185 (C vs. DR) *p* > 0.9999 (C vs. AMD) *p*=0.0072^*∗*^ (AMD vs. DR)
Baseline IGF-1 (median/mean ± SD (pg/ml))	35/37 ± 12.8	57/65 ± 32.1	*p*=0.0056^*∗*^	49/54 ± 18.5	*p*=0.0039^*∗∗*^ (KW) *p* > 0.9999 (C vs. DR) *p*=0.0312^*∗*^ (C vs. AMD) *p*=0.0056^*∗*^ (AMD vs. DR)
IQR	24–51	37–99		43–61	
Baseline VEGF-A (median/mean ± SD (pg/ml))	43/42 ± 16	53/75 ± 57.01	*p* > 0.2077	61/87 ± 78.8	*p*=0.0751 (KW) *p*=0.9999 (C vs. DR) *p*=0.1075 (C vs. AMD) *p* > 0.2077 (AMD vs. DR)
IQR	30–57	35–117		38–112	

AMD: age-related macular degeneration; DR: diabetic retinopathy; C: control; VEGF-A: vascular endothelial growth factor-A; IGF-1: insulin-like growth factor-1; FT: Fisher's exact test; KW: Kruskal–Wallis test; CS: chi-square test; ns: not significant; *n*: number; SD: standard deviation.

**Table 2:** Systemic VEGF-A and IGF-1 concentrations.

	AMD	PDR	NPDR	*p* (AMD vs. DR)
VEGF-A (median/mean (pg/ml)) at B (IQR)	43/43 (30–57)	54/73 (29–138)	52/77 (31–109)	*p*=0.0798 (MW)
VEGF-A (median/mean (pg/ml)) 1 week (IQR)	8/9 (8–8)	3/4 (0–7)	2/2 (1–3)	*p* < 0.0001^*∗∗∗*^ (MW)
VEGF-A (median/mean (pg/ml)) 4 weeks (IQR)	17/20 (8–25)	14/13 (9–19)	20/20 (13–24)	*p*=0.6777 (MW)
*p*	*p* < 0.0001^*∗∗∗*^ (F) *p* < 0.0001^*∗∗∗*^ (B vs. 1 week) *p*=0.0054^*∗*^ (B vs. 4 weeks)	*p* < 0.0001^*∗∗∗*^ (F; DR over time) *p* < 0.0001^*∗∗∗*^ (B vs. 1 week) *p*=0.0327^*∗*^ (B vs. 4 weeks)		

IGF-1 (median/mean (pg/ml)) at B (IQR)	35/37 (24–51)	83/82 (48–117)	54/59 (29–88)	*p*=0.0071 (MW)
IGF-1 (median/mean (pg/ml)) 1 week (IQR)	41/38 (27–45)	71/129 (45–243)	43/48 (29–66)	*p*=0.0639 (MW)
IGF-1 (median/mean (pg/ml)) 4 weeks (IQR)	41/39 (28–48)	82/98 (34–181)	40/51 (34–58)	*p*=0.3288 (MW)
*p*	*p*=0.3287 (F) *p*=0.5834 (B vs. 1 week) *p*=0.3359 (B vs. 4 weeks)	*p*=0.3050 (F; DR over time) *p* > 0.9999 (B vs. 1 week) *p*=0.3146 (B vs. 4 weeks)		

AMD: age-related macular degeneration; DR: diabetic retinopathy; PDR: proliferative diabetic retinopathy; NPDR: nonproliferative diabetic retinopathy; VEGF-A: vascular endothelial growth factor-A; IGF-1: insulin-like growth factor-1; B: baseline; MW: Mann–Whitney test; F: Friedman test.

## Data Availability

The data that support the findings of this study are available from the corresponding author upon reasonable request.
